# Anatomical Theories of the Pathophysiology of Cancer-Related Lymphoedema

**DOI:** 10.3390/cancers12051338

**Published:** 2020-05-23

**Authors:** Hiroo Suami

**Affiliations:** Australian Lymphoedema Education, Research and Treatment Program, Faculty of Medicine, Health and Human Sciences, Macquarie University, Sydney, NSW 2109, Australia; hiroo.suami@mq.edu.au; Tel.: +61-2-98502359; Fax: +61-2-98502701

**Keywords:** anatomy, lymphatic system, cadaver, indocyanine green, lymphangiogenesis, regression

## Abstract

Lymphoedema is a well-known concern for cancer survivors. A crucial issue in lymphoedema is that we cannot predict who will be affected, and onset can occur many years after initial cancer treatment. The variability of time between cancer treatment and lymphoedema onset is an unexplained mystery. Retrospective cohort studies have investigated the risk factors for lymphoedema development, with extensive surgery and the combination of radiation and surgery identified as common high-risk factors. However, these studies could not predict lymphoedema risk in each individual patient in the early stages, nor could they explain the timing of onset. The study of anatomy is one promising tool to help shed light on the pathophysiology of lymphoedema. While the lymphatic system is the area least investigated in the field of anatomical science, some studies have described anatomical changes in the lymphatic system after lymph node dissection. Clinical imaging studies in lymphangiography, lymphoscintigraphy and indocyanine green (ICG) fluorescent lymphography have reported post-operative anatomical changes in the lymphatic system, including dermal backflow, lymphangiogenesis and creation of alternative pathways via the deep and torso lymphatics, demonstrating that such dynamic anatomical changes contribute to the maintenance of lymphatic drainage pathways. This article presents a descriptive review of the anatomical and imaging studies of the lymphatic system in the normal and post-operative conditions and attempts to answer the questions of why some people develop lymphoedema after cancer and some do not, and what causes the variability in lymphoedema onset timing.

## 1. Introduction

Lymphoedema is a well-known side-effect of post-cancer treatment. The standard treatment for lymphoedema includes the daily wearing of a compression garment on the affected body part to prevent the progression of the disease [1]. Management recommendations are normally provided by an allied health professional who is accredited as a lymphoedema therapist. Surgical interventions can be employed to reduce patient discomfort, but they cannot provide a cure for lymphoedema at present [2,3]. Unlike cancer, lymphoedema is not a life-threatening condition, but its impact is great, causing life-long physical, psychological and economic distress for those who suffer from it [4].

One of the crucial issues in lymphoedema is that oncologists cannot predict which patients will suffer from the disease in the future and which will be spared. Lymphoedema can develop at any time from six months to many years after completion of cancer treatment, and, thus, the onset is variable [5]. Although cancer survivors are relieved to have their cancer cured, they are still under threat of possibly developing lymphoedema afterwards. Retrospective cohort studies have investigated the risk factors for lymphoedema and have identified extensive surgery that includes radical lymph node dissection, the combination of radiotherapy and surgery, and obesity as three high-risk factors [6,7,8]. The results of these studies helped to provide a ratio of odds for lymphoedema development, but they are of no help to patients at an individual level.

The study of lymphatic anatomy is considered to be one of the promising tools to help explain the pathophysiology of lymphoedema. The lymphatic system is a part of the vascular system, but it has been far less studied than the blood circulatory system. The role of the lymphatic system is to collect and transport interstitial tissue fluid and protein substances that have escaped from the blood capillaries. The dysfunction of the lymphatic system causes oedema in the affected regions. Cancer cells also travel via the lymphatic system, and the process of cancer cells reaching the regional lymph nodes and taking root there is known as lymphogenous metastasis. Hence, the lymphatic system is commonly targeted in cancer treatment to prevent the disease from spreading. In this way, cancer treatment is a direct cause of lymphoedema.

Previous anatomical studies have focused on the development of a lymphoedema model in animals and patients affected by lymphoedema, but less attention has been paid to patients who survived cancer and did not develop lymphoedema afterwards [9,10,11,12]. I speculate that the anatomy of the lymphatics changes in all patients after cancer treatment, but for the majority of them, those changes do not reach the threshold for developing into lymphoedema. Recently, near-infrared indocyanine green (ICG) fluorescence lymphography has been widely used to provide new imaging data for the assessment of lymphoedema [13,14,15,16,17].

This article presents a review of studies into the anatomical changes that occur in the lymphatic system post-lymph node dissection and discusses anatomical theories as to why some patients develop lymphoedema and others do not.

## 2. Normal Lymphatic Anatomy

**Figure 1:**
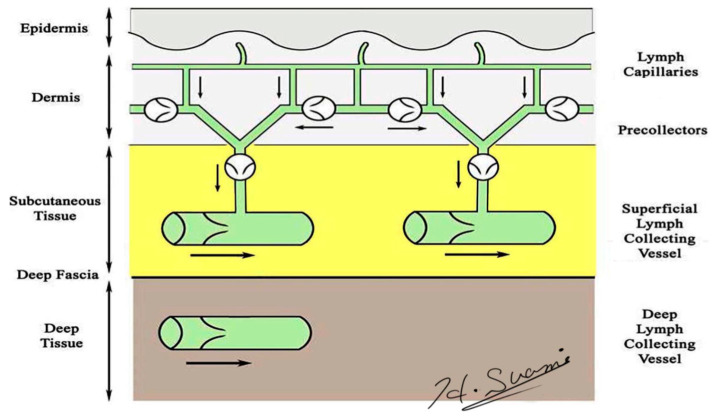
Schematic diagram of the lymphatic system. (Reproduced with the permission of Hiroo Suami).

Precise knowledge of normal lymphatic anatomy provided the baseline information needed to identify the changes to lymphatic structures resulting from lymph node dissection. Lymphatic vessels originate as avalvular lymphatic capillaries in the superficial dermis and connect to valvular pre-collectors in the deep dermis. Pre-collectors merge with each other and change course to run vertically from the skin to the deep layer. These vertical vessels connect to the superficial lymph-collecting vessels running horizontally in the subcutaneous layer. The deep lymph-collecting vessels run alongside the major arteries below the deep fascia (Figure 1) [18,19]. The perforating lymphatic vessels are branches of the deep lymph-collecting vessels that run along the perforating arteries above the deep fascia. For example, breast cancer can metastasize to the internal mammary lymph nodes via the perforating lymphatic vessels. Both the superficial lymph-collecting vessels and the perforating lymphatic vessels exist in the subcutaneous fat layer above the deep fascia, but there are no connections between them. In general, the superficial and deep lymph-collecting vessels are independent of each other, and they only unite in the proximal body region, such as level-two nodes in the axilla or intrapelvic nodes. 

**Figure 2:**
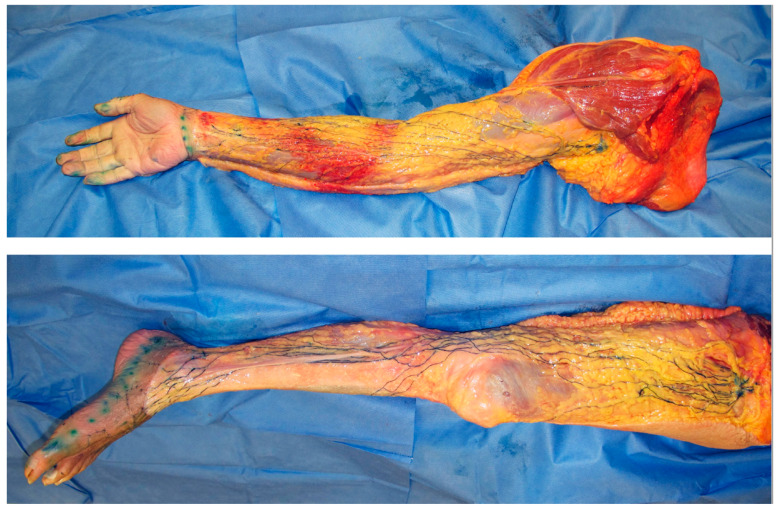
Dissection photos of the superficial lymph-collecting vessels and their corresponding lymph nodes in the upper and lower limbs visualized using the microinjection technique. The lymphatic vessels were injected with blue acrylic dye, and the skin and adipose tissue were removed just above the vessels. Arrows indicate the sentinel node/s of the upper limb (white) and lower limb (black).

In lymphoedema, excess lymph fluid and proliferation of adipose tissue occur predominantly in the subcutaneous fat layer above the deep fascia [20,21]. These findings indicate that dysfunction of the superficial lymphatic system can be considered a major cause of lymphoedema. The superficial lymph-collecting vessels are independent of each other and may diverge and converge at various points. Interconnections between vessels are uncommon and, therefore, do not form a network (Figure 2) [18]. 

**Figure 3:**
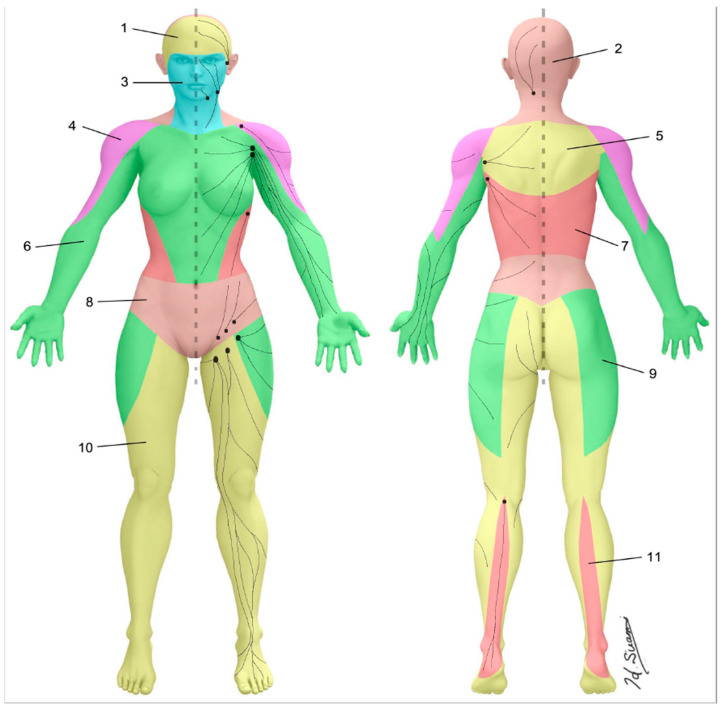
Lymphosomes of the body. The lymphatic territories are demarcated according to their corresponding lymphatic basins: (1) temporal, (2) occipital, (3) submental, (4) subclavicular, (5) subscapular, (6) lateral axillary, (7) pectoral, (8) superior inguinal, (9) lateral inguinal, (10) inferior inguinal, (11) popliteal. (Reproduced with permission of Hiroo Suami).

The skin can be divided into superficial lymphatic territories called “lymphosomes” (Figure 3) [22,23]. Both the upper and lower limb are demarcated into two lymphosomes, one dominant and one supplementary. In the upper limb, the dominant lymphatic territory drains to the axillary nodes. The supplementary territory covers the lateral upper arm. It bypasses the axillary nodes and drains to the supraclavicular nodes [24,25]. In the lower limb, the dominant lymphatic territory drains to the superficial inguinal nodes. The supplementary territory covers the calf region and drains to the popliteal node on its way to the deep inguinal lymph nodes via the deep lymph-collecting vessels in the thigh [26,27].

There are two types of lymph nodes, known as regional and interval nodes. The axillary and inguinal nodes are regional nodes, and the popliteal and epitrochlear nodes are interval nodes [28]. The difference between regional and interval nodes is demonstrated by the number of afferent lymphatic vessels that connect to them. Multiple afferent lymphatic vessels connect to the regional nodes, but only one or two afferent vessels connect to interval nodes.

The focus in lymphoedema is generally the lymphatics of the upper or lower limb, and the torso is often overlooked. The lymphatics in the torso are divided by the sagittal midline and horizontal line at the umbilical level [29]. Each quadrant drains to the ipsilateral axillary or inguinal lymph nodes. The superficial lymph-collecting vessels in the torso are also affected by lymph node dissection because the axilla and inguinal regions are not the only regions to which drainage from the limbs occurs.

## 3. Anatomical Changes in Lymphoedema

### 3.1. Dermal Backflow

The presence of dermal backflow is a specific imaging criterion for the diagnosis of lymphoedema. This indicator was originally used in lymphangiography [30]. When radiocontrast was injected into a superficial lymph-collecting vessel in the dorsal hand or foot in patients with lymphoedema, the injected contrast overflowed to fill the dermal lymphatics. The identification of dermal backflow has also been utilized in lymphoscintigraphy and ICG lymphography to diagnose lymphoedema [13,14,15,16,17,31].

**Figure 4:**
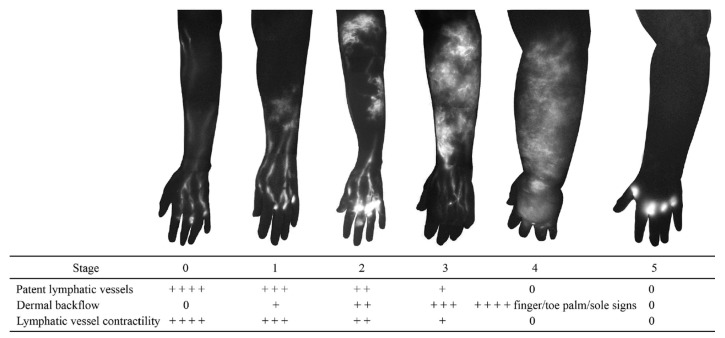
Indocyanine green lymphography staging scale. Stage 0 represents normal lymphatics, and stages 1 to 5 represent increasing lymphoedema severity. There is no dye movement at Stage 5. (Reproduced from [35] with permission).

Dermal backflow is composed of dilated dermal lymphatic capillaries and pre-collectors [32]. It develops when the obstruction of the superficial lymph-collecting vessels causes the valves in the pre-collectors to become incompetent. Dermal backflow is considered to be the body’s anatomical response to lymphatic obstruction by creating alternative pathways to transport lymph fluid [33]. The amount of dermal backflow in the limbs correlates with the severity of lymphoedema (Figure 4) [34,35].

**Figure 5:**
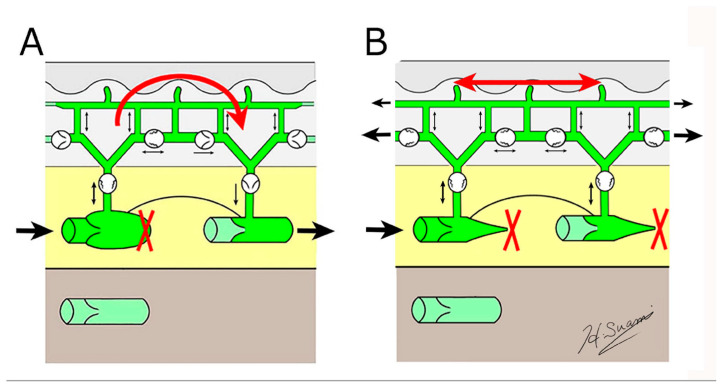
Schematic diagrams showing the structural changes in lymphoedema. (**A**) dermal backflow forms a bridge between obstructed and patent lymphatic vessels, and (**B**) dermal backflow serves as a substitute route to transport lymph fluid following the obstruction of the superficial collecting vessels through fibrosis. (Reproduced with permission of Hiroo Suami).

Dermal backflow has two functions in regulating lymph transport in lymphoedema (Figure 5). 

Firstly, if patent, superficial lymph-collecting vessels remain in the affected limb, it bridges the gap between the non-patent and patent vessels. Secondly, if all the superficial lymph-collecting vessels have deteriorated, the dermal backflow becomes the only pathway to drain lymph fluid from the affected area. 

The lymph capillaries and pre-collectors in the dermis are several times smaller than the lymph-collecting vessels [19], so if dermal backflow intervenes in the process of lymphatic drainage, lymph flow is restricted. Consequently, dermal backflow is considered a negative pathological change in lymphoedema because patients feel discomfort, and it causes skin induration in the affected area due to restricted flow. However, in anatomical terms, dermal backflow is a positive change as it enables lymph drainage to be maintained by creating an alternative pathway. 

### 3.2. Lymphangiogenesis

**Figure 6:**
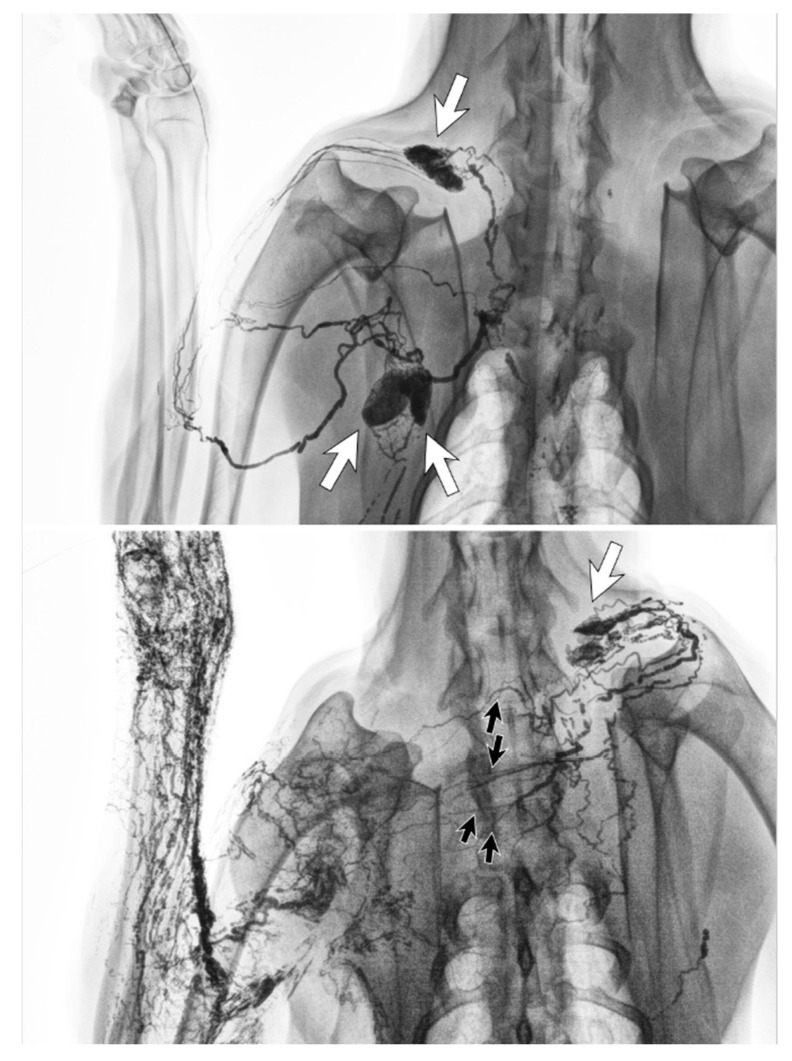
Lymphangiography images of a canine before lymph node dissection surgery (**top**) and six months afterwards (**bottom**). The lymphatic vessels from the left forelimb crossed the front midline and connected to the right cervical lymph node (white arrow) via regenerated lymphatic vessels (black arrows). (Reproduced from [36] with permission.).

The lymphatic system can regenerate after lymph node dissection. Animal studies found that although lymph nodes themselves did not regenerate, the lymphatic vessels could regenerate to develop new lymph drainage pathways (Figure 6) [36,37]. The types of vessels able to regenerate were both the fine lymphatic capillaries and also the larger lymph-collecting vessels. In these studies, lymphangiogenesis started at the site of severed, afferent lymphatic vessels, with the newly regenerated vessels connecting to the remaining lymph nodes to maintain the lymph drainage pathways. 

**Figure 7:**
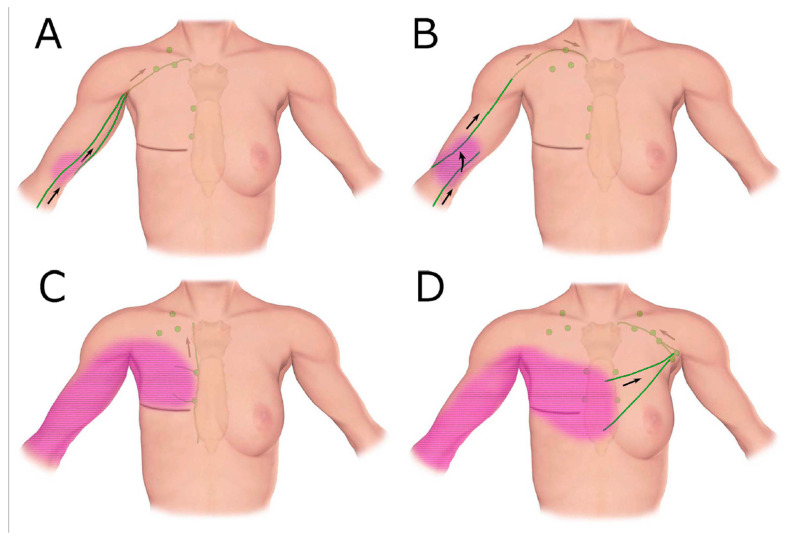
Schematic diagrams show patterns of lymphatic drainage in upper extremity lymphoedema. (**A**) The ipsilateral axillary region, (**B**) the clavicular region, (**C**) the parasternal region, (**D**) The contralateral axillary region. (Reproduced from [42] with permission.).

Clinical imaging studies using lymphangiography, lymphoscintigraphy and ICG lymphography also found that the lymphatic system in the affected limb could reconnect to the remaining lymph nodes through lymphangiogenesis [38,39,40,41,42]. The location of the remaining nodes varied from the ipsilateral axillary, supraclavicular, internal mammary and contralateral axillary nodes (Figure 7) [42].

### 3.3. Detour via the Deep Lymphatic System 

When a patient undergoes lymph node dissection and does not develop lymphoedema later, the common assumption is that a part of the original lymphatic pathway is intact. For example, breast surgeons do not skeletonize the axillary vein during axillary node dissection, because anecdotal experience suggests an increasing chance of lymphoedema development. However, anatomical studies indicate that both the breast and arm sentinel nodes were located in level-one nodes in the lateral axillary region [43]. Although the deep lymph-collecting vessels may be preserved by the remaining soft tissue around the axillary vein, axillary node dissection always creates a surgical gap in the lymphatic pathway from the arm. This suggests that there is another mechanism at play that is preventing the development of lymphoedema in these patients.

**Figure 8:**
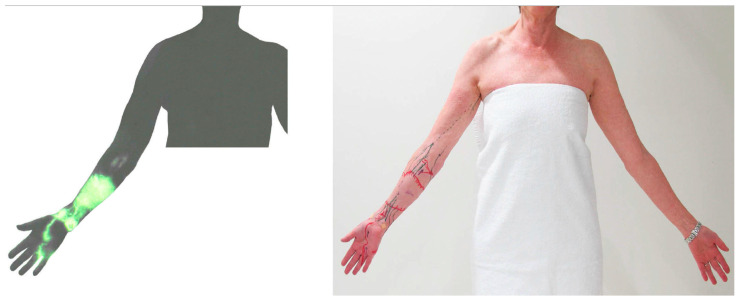
Indocyanine green (ICG) lymphography image (**left**) and tracing photo (**right**) in a patient with breast cancer-related lymphoedema. The ICG signal disappeared at the elbow and reappeared in the axilla. This suggests a connection between superficial and deep lymphatics.

We have previously reported that the superficial and deep lymph-collecting vessels are independent of each other in normal anatomy [18,19]. However, post-operative lymphatic anatomy changes significantly from the original anatomy to maintain lymphatic drainage pathways. ICG lymphography in some lymphoedema patients identified that the superficial lymph-collecting vessels connected to the deep lymph collecting vessels at the medial elbow (Figure 8). A cadaver study using post-axillary node dissection also found the same unusual connection [28]. It appears that the preservation of the deep lymphatic pathway during axillary node dissection may help to establish connections between the superficial and deep lymphatic systems and serve as a detour route to prevent the progression of lymphoedema.

### 3.4. Detour via the Lymphatics in The Torso

The lymphatic system in the torso can be affected by lymph node dissection, with either breast or abdominal oedema identified. Newer techniques have been developed to reduce the extent of breast surgery, and breast-conserving therapy is becoming a more popular option in early breast cancer [44]. Breast-conserving therapy and oncoplastic surgery can achieve better cosmetic outcomes [45]. However, combined lymph node dissection and radiation therapy compromises lymphatic drainage from the remaining breast tissue and gives rise to the possibility of developing breast oedema [46].

**Figure 9:**
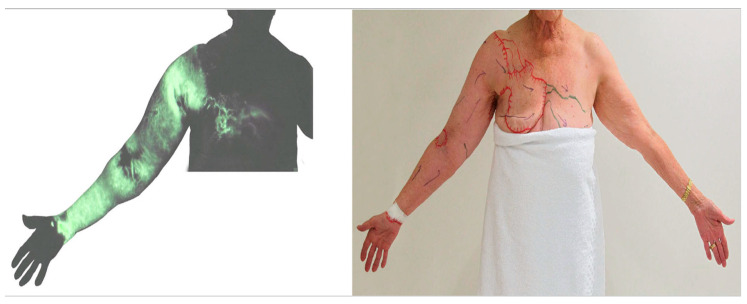
ICG lymphography image (**left**) and tracing photo (**right**) in a patient with breast cancer-related lymphoedema. Dermal backflow in the right arm extended to the right front chest and then connected to the left axillary lymph node via the superficial lymph-collecting vessels in the left chest. (Reproduced from [17] with permission).

Breast and abdominal oedema can be independent of, or associated with, lymphoedema in the limbs. In some cases of advanced breast cancer-related lymphoedema, dermal backflow covered the whole arm and extended to the front chest. The tracer drained to the internal mammary lymph nodes via the perforating lymphatic vessels or crossed the front sagittal midline and drained to the contralateral axillary nodes via normal lymphatics in the unaffected breast (Figure 9) [17].

In these cases, the lymphatic system in the torso worked as a collateral lymphatic drainage pathway from the affected arm. Similar anatomical changes were observed in leg lymphoedema. Advanced unilateral leg lymphoedema sometimes drained to the contralateral inguinal nodes via the lymphatics in the lower abdomen. The possible interaction of the lymphatic system between the limbs and torso should be considered in post-lymph node dissection because limb lymphoedema can cause breast and abdominal oedema.

## 4. Anatomical Theories of Cancer-Related Lymphoedema

Knowledge of normal lymphatic anatomy and an understanding of how it changes post-operatively may shed light on the pathophysiology of lymphoedema. Olszewski conducted lymphangiography studies to investigate post-operative lymphoedema in animal models [47]. His results found that post-operative lymphatic anatomy was significantly changed from the original anatomy, although clinical lymphoedema did not develop immediately, or sometimes at all. When lymphoedema developed after surgery, the time from anatomical structural change to the onset of clinical symptoms took from a minimum 7–8 months to several years in some cases. The anatomical changes included dilatation of both dermal lymphatics and lymph-collecting vessels. Below, this manuscript will describe a framework for the different stages of lymphatic vessel change after lymph node dissection.

### 4.1. Lymph Node Dissection

When axillary lymph node dissection is performed, the superficial lymphatic drainage pathway from the arm is interrupted and a surgical break is created because arm sentinel nodes are located in level-one nodes in the lateral axilla [18]. The deep lymphatic system may also be interrupted when level-two nodes are removed. If the patient has supplementary drainage pathways to the supraclavicular node in the lateral upper arm, they will be outside the sphere of dissection and will be able to keep draining lymph fluid [24,25].

When the break occurs, lymph fluid leaks from the stump of the lymph-collecting vessels, and the surgical drains remove the pooled fluid in the defective tissue. Lymphoedema does not develop immediately because excess fluid is removed from the wound.

### 4.2. Lymphangiogenesis

Lymphangiogenesis occurs during the wound-healing process to restore the break caused by surgery. New lymphatics are regenerated from the distal stumps of the arm lymph-collecting vessels and connect to the remaining lymph nodes [36]. The patient may notice some arm swelling, but the symptoms gradually subside. The initial process to restore lymphatic pathways in this way may take around one month [47].

### 4.3. Latent Phase

The regenerated lymphatics develop from immature vessels into stable, mature vessels. If this process is successful, lymphoedema may not develop at all. Scar formation, delayed wound-healing with seroma, post-operative irradiation, and cellulitis are the factors that are considered to adversely impact the maturation process and may cause regression of the immature vessels.

### 4.4. Development of Lymphoedema

If the temporarily restored lymphatics lose function, lymphoedema will develop. Obstruction of the superficial lymph-collecting vessels causes incompetent valves to form in the pre-collectors, resulting in dermal backflow, forming a connection between patent and non-patent lymph-collecting vessels [32,42]. If the deep lymph-collecting vessels are intact, the obstructed, superficial lymph-collecting vessels may create a detour to the deep lymphatics (Figure 8). The previous dominant pathway to the axilla may still work, but the restriction of lymph flow will cause dermal backflow in the distal arm. Alternative pathways to the other lymph nodes will develop to maintain lymph drainage in the lymphoedematous limb (Figure 9) [42].

Olszewski’s study found that the gap in time between lymph node dissection and lymphoedema development may be explained by the length of time taken for the initial bridging process through lymphangiogenesis to occur, followed by the regression of the regenerated lymphatics [47]. Akita’s prospective cohort study of ICG lymphography in breast cancer patients found that dermal backflow could be identified in patients in the latent phase of lymphoedema, where no clinical symptoms were evident [48]. This study suggested that ICG lymphography could be used to screen for breast cancer-related lymphoedema at the subclinical stage.

Several ideas have been proposed to prevent the development of lymphoedema. One is the vascular endothelial growth factor C (VGEF-C) drug that has been developed to the clinical trial stage [49]. Another is Boccardo’s creation of a microsurgical lymphovenous bypass during axillary dissection to protect the lymph drainage pathways from the arm and promote healing [50]. A third approach is a synthetic fibrous thread called a “BioBridge” that has been developed to facilitate the process of lymphatic regeneration [51]. These new procedures may prevent the regression of newly regenerated lymphatic vessels during the latent phase.

The exact factors that determine which patients will develop lymphoedema after cancer treatment have not yet been identified. However, medical imaging plays an important role in screening patients before lymphoedema develops, and techniques to do this are advancing all the time. Anatomical theories are also vital in promoting understanding about what structural changes occur post-surgery and how these changes correlate with lymphoedema development.

## 5. Conclusions

Lymphoedema is a debilitating condition that affects many people who have survived cancer. However, we are still unable at present to identify who among them will develop lymphoedema and who will not. This paper has reported on the anatomical changes that occur after surgery and how they contribute to the maintenance of lymphatic drainage pathways. We have also described how the mechanism of lymphangiogenesis works to bridge the gap in the lymphatics caused by surgery and demonstrated that regression of the immature lymphatic vessels may explain the delayed onset of cancer-related lymphoedema.
